# Real-Time Cutting Temperature Monitoring and Tool Wear Prediction with Integrated Thin-Film Thermocouples and Coupled Simulation

**DOI:** 10.3390/mi17060693

**Published:** 2026-06-04

**Authors:** Yingyuan Luo, Fenghao Zuo, Binghai Lyu, Xueliang Zhang, Xianfan Ge

**Affiliations:** 1 School of Intelligent Manufacturing, Hangzhou Polytechnic, Hangzhou 311402, China; 2 School of Information Engineering, Henan University of Science and Technology, Luoyang 471023, China; 17839769933@163.com; 3 Hangzhou Runde Wheel Manufacturing Co., Ltd., Hangzhou 311407, China; icewater7812@126.com; 4 Shanghai Waigaoqiao Shipbuilding Co., Ltd., Shanghai 200137, China

**Keywords:** thin-film thermocouple, cutting temperature, finite element simulation, tool wear, archard model, cryogenic cooling

## Abstract

Accurate measurement of the temperature in the cutting zone is essential for closed-loop machining. However, it remains difficult due to the small size of the tool–chip contact area, its partial concealment by chips and the steep thermal gradients present. This study presents an integrated framework that combines a thin-film thermocouple (TFTC) on the rake face of a polycrystalline cubic boron nitride (PCBN) tool with a thermo-mechanical wear-coupled simulation in order to monitor cutting temperature and predict tool wear. The three-dimensional finite-element turning model includes a moving heat source that represents plastic and frictional heat at the tool–chip interface, as well as an Archard-type wear law that is enhanced by a temperature correction factor. The TFTC is fabricated by magnetron sputtering NiCr and NiSi films onto an insulating layer, after which it is embedded in the tool as a minimally intrusive in situ sensor. Turning experiments on AISI 1045 steel were performed at spindle speeds of 1000–3000 rpm, feeds of 0.05–0.20 mm/rev and depths of cut ranging from 0.3 to 1.0 mm under dry, wet (emulsion) and cryogenic (liquid nitrogen) cooling conditions. Simulated temperature fields reveal strong localisation at the tool–chip contact and a nonlinear increase in peak rake-face temperature with spindle speed, which fits a quadratic regression with R^2^ = 0.99. The TFTC shows a response time of around 0.3 s with less than 5% overshoot, and its thermoelectric voltage is almost perfectly linear with temperature (R^2^ = 1), with a sensitivity of approximately 12 µV/°C. During cutting, TFTC readings agree with infrared measurements within ±3 °C and demonstrate improved robustness in occluded zones. The coupled wear model replicates the observed wear growth trend with the compact expression VB = 0.0001·t^0.8^. Sensitivity tests indicate that thermo-mechanical coupling increases wear rates compared to single-factor models, and that cooling reduces thermal loads by approximately 15% (wet) and 25% (cryogenic).

## 1. Introduction

The growing demand for high precision, efficiency and tool life in modern manufacturing is pushing machining processes to their limits. At the heart of these processes is the cutting zone, a microscopic region of intense activity where material is removed under extreme conditions. The most critical process variable is cutting temperature, which has a profound influence on chip formation, workpiece surface quality, dimensional accuracy, metallurgical changes and, most importantly, tool life [1,2]. Heat is primarily generated by plastic deformation and friction at the tool–chip interface [3,4]. As machining speeds increase and the use of difficult-to-machine materials such as aerospace alloys and hardened steels grows, thermal loads near the cutting edge become increasingly severe, with temperatures soaring to hundreds or even thousands of degrees Celsius [5]. This localised high temperature primarily drives accelerated thermal wear, damage to tool coatings, altered friction characteristics and the generation of thermal stress [6,7]. Therefore, accurately monitoring and predicting cutting temperatures is critical for understanding and optimising the process, and for realising closed-loop smart manufacturing [8], particularly in enhancing tool life prediction and overall process control.

Despite continuous technological advancements, accurately and locally measuring temperatures in real time within the dynamic and harsh cutting zone remains extremely challenging [9]. Traditional contact methods, such as embedded thermocouples, have slow response times and high thermal inertia. Furthermore, due to size limitations, they struggle to capture the temperatures of the hottest hotspots near the cutting edge [10]. Non-contact methods, such as infrared thermometers, are severely limited by obstructions to the line of sight (e.g., chips), uncertainties in material emissivity and reflective interference from hot chips or workpieces. This makes it difficult to achieve continuous and reliable measurements during actual machining [11,12]. These limitations have driven the search for alternative, more robust sensing technologies. Thin-film thermocouples (TFTCs) offer a significant solution to the aforementioned measurement challenges due to their ultra-thin, miniaturised, fast-response and low thermal inertia characteristics [13,14]. They can be deposited directly onto the tool surface or an insulating layer, enabling highly localised temperature measurement and reducing the impact of chip obstruction. However, applying TFTCs to harsh cutting environments still presents unique challenges, such as chip abrasion, severe thermal cycling and mechanical vibration. Careful design of material selection (e.g., NiCr/NiSi, Pt/Rh), junction temperature optimisation and protective coatings are required to ensure sensor durability and signal integrity [15,16].

In addition to temperature monitoring, accurately predicting tool wear is essential for optimising production schedules, reducing costs and ensuring product quality. While traditional wear models, such as the Taylor life equation [17], are practical in industrial applications, they lack insight into the mechanisms of wear. The Archard model offers a more physically grounded understanding of wear [18]; however, it assumes a constant wear coefficient, which is overly simplistic in high-temperature machining environments, particularly when wear coefficients vary due to changes in adhesive wear, energy dissipation and chemical reactions [19]. Importantly, high temperatures can significantly accelerate tool degradation by enhancing diffusion between the tool and workpiece, accelerating oxidation reactions, and promoting softening of the binder in composite tools (e.g., PCBN) [20,21]. This highlights the importance of developing temperature-aware wear models that incorporate temperature-dependent wear coefficients or explicitly link wear phenomena to local temperature fields, thereby improving predictive accuracy.

Thermomechanical finite element (FE) simulation has become a powerful tool for analysing cutting processes, capable of providing detailed predictions of temperature, stress and strain distributions [22,23]. However, its accuracy depends heavily on precise friction and heat transfer parameters, and it is often disconnected from real-time process monitoring, which limits its ability to adapt to dynamic changes [24]. Therefore, there is an urgent need for an integrated framework that combines real-time sensing with predictive thermomechanical wear simulation. Such a collaborative system could significantly improve the accuracy of wear prediction and enable advanced process control and optimisation based on real-time process conditions. Furthermore, optimising cooling strategies is a critical application of thermal management. Quantifying the effects of different cooling methods (e.g., dry cutting, wet cutting, and advanced cryogenic cooling such as liquid nitrogen) on cutting zone temperature and wear rates is crucial for making informed decisions [25,26].

This thesis focuses on the joint design and validation of sensors and models. The primary objectives are:

(1) To develop a three-dimensional thermomechanical finite element (FE) turning model that incorporates a moving heat source and Coulomb friction.
(2) To extend this model to include an Archard-type wear law with temperature correction factors. To fabricate and integrate a novel, highly responsive nickel–chromium/nickel–silicon (NiCr/NiSi) thin-film thermocouple (TFTC) that is specifically designed for precise, localised, real-time, in situ temperature monitoring in harsh cutting environments and to comprehensively characterise its static and dynamic responses.
(3) To conduct turning experiments under dry, wet and low-temperature cutting conditions and validate the simulated temperatures using TFTC and infrared (IR) measurements, demonstrating the accuracy of the TFTC’s in situ monitoring capabilities.
(4) To establish quantitative relationships between cutting parameters, cutting temperatures and rake face wear (VB) and to explore design directions that improve sensor robustness and lifespan. This will ultimately enhance tool wear prediction through an integrated framework.

The remainder of this paper is organised as follows: Section 2 reviews relevant work on cutting temperature measurement, TFTC technology and wear modelling. Section 3 introduces the coupled simulation framework, the fabrication and calibration of the TFTC and the experimental setup. Section 4 presents simulation and experimental results focusing on temperature fields, TFTC responses and wear prediction. Section 5 concludes the paper.

## 2. Related Work

### 2.1. Cutting Temperature Measurement in Machining

The cutting temperature has been measured using various conduction and radiation methods. Reviews consistently highlight the practical trade-offs among spatial resolution, bandwidth, robustness, and process disturbance [27]. Embedded thermocouples are frequently used in research and production diagnostics, but their limitations include the need to place the junction and the requirement to modify the tool or holder. Natural thermocouple approaches, which use the tool–workpiece as the thermocouple, can provide the average interface temperature but require electrical isolation and are sensitive to chip-induced short circuits. Radiation methods offer contactless measurement but are limited by emissivity uncertainty and chip obstruction, especially during turning and drilling.

Kus et al. performed simultaneous measurements with a K-type thermocouple and an IR pyrometer, highlighting that in situ IR measurements are susceptible to chip blockage and require careful emissivity management [28]. These findings support the use of embedded sensors that can function at the cutting edge without optical access.

### 2.2. Thin-Film Thermocouples for Harsh Environments and Machining

TFTCs enable miniaturised junctions and low thermal inertia through thin-film deposition and micro-patterning. An open-access review in iScience surveys flexible TFTC structures, materials, and fabrication methods, highlighting opportunities for wide-range sensing and multi-sensor integration [29]. For harsh environments, ceramic and oxide systems have been studied because they can sustain thermoelectric stability at high temperatures. Xie et al. reported ITO/In_2_O_3_ TFTCs deposited on Al_2_O_3_ substrates and confirmed their response via pulsed-laser calibration, demonstrating the feasibility of transient high-temperature sensing [30].

In machining applications, the sensor must also be able to withstand abrasion, thermal cycling and mechanical vibration. To meet these requirements, protective layers, optimised junction placement and substrate designs that reduce thermal stress are used. Thin-film thermocouples produced by magnetron sputtering (including NiCr/NiSi systems) strike a good balance between manufacturing complexity and performance, and have been used for various in situ temperature monitoring tasks.

### 2.3. Wear Modelling and Temperature Dependence

Archard-type models remain widely used because they link wear volume to load, sliding distance, and hardness through a wear coefficient. The traditional wear law for unlubricated contacts and its mechanistic interpretation were established in foundational tribology research [31]. However, recent studies highlight that the wear coefficient is not constant across different conditions, especially when adhesive wear transitions and energy dissipation change. Choudhry et al. proposed an energy-based interpretation of the Archard wear coefficient and demonstrated that it can vary spatially and with accumulated deformation energy, explaining why calibration is necessary when using Archard-type models in complex contacts [32].

In machining, additional complexities stem from oxidation, diffusion, and chemical reactions at high temperatures. For PCBN tools, thermodynamic analysis shows that diffusion and oxidation wear increase with temperature and are influenced by the workpiece material chemistry [33]. This encourages temperature-aware wear modelling, either by applying temperature-dependent wear coefficients or by linking wear to local temperature fields.

### 2.4. Multi-Physics Simulation and Sensor Co-Design

Thermo-mechanical simulation of cutting has advanced significantly and can accurately reproduce chip morphology and temperature trends when constitutive models and friction are correctly identified. An open-access numerical–experimental study on orthogonal cutting of AISI 1045 steel demonstrates the importance of simultaneously validating forces and temperature-related outputs to calibrate friction and enhance predictive accuracy [34]. When used with embedded sensors, simulation can also help determine optimal sensor placement and assess the extent to which the sensor disturbs heat flow.

In summary, the literature highlights three key points:

(1) Accurate, localised temperature measurement remains challenging in actual cutting zones.
(2) TFTCs present a practical, embedded solution when properly protected and calibrated.
(3) Temperature-aware wear modelling benefits from integrating spatial temperature fields. These points motivate the integrated methodology outlined below.

## 3. Materials and Methods

### 3.1. Overview

The proposed system comprises a thermo-mechanical-wear coupled finite element (FE) model and an embedded NiCr/NiSi thermo-friction contact (TFTC) sensor on the rake face of a polycrystalline cubic boron nitride (PCBN) insert. The simulation predicts transient temperature fields and contact conditions, while the TFTC provides local temperature measurements to validate and calibrate the heat partition and wear parameters.

### 3.2. Governing Equations and Interface Laws

Heat transfer in the tool–chip–workpiece system is described by transient conduction with internal heat generation:

(1) $$\rho c_{p}\partial T/\partial t=\nabla \cdot \left(k\nabla T\right)+Q˙$$

where *Q* is density, *c* is specific heat and *k* is thermal conductivity. *Q*(*x*, *t*) represents volumetric heat generation. Heat is produced by plastic deformation in the primary shear zone and by frictional sliding at the tool–chip interface. To accurately represent the localisation and movement of heat input, a moving heat source model is employed, in which the heat source centroid moves along the contact length as the chip advances.

At the tool–chip interface, friction is modelled using Coulomb’s law.

(2) $$\tau_{f}=\mu p_{n},{q˙}_{f}=\eta \tau_{f}v_{s}$$

where $\tau_{f}$ is frictional shear stress, *μ* is the friction coefficient, and *p* is the contact pressure. The frictional heat flux is estimated as, where $v_{s}$ is the sliding velocity, and *η* is the fraction of mechanical work converted to heat. Heat partition between the tool and the chip is treated via boundary conditions; partition coefficients are adjusted to match the embedded temperature measurement.

### 3.3. Wear Model and Temperature Correction

Tool wear is described using an Archard-type law:

(3) $$dV_{w}/ds=k\left(T\right)\cdot \left(W/H\right)$$

where *dV/ds* is the incremental wear volume per sliding distance, *W* is the normal load, *H* is the hardness and *k*(*T*) is the effective wear coefficient. In machining, *k*(*T*) captures the combined effects of temperature and the working environment. In this study, we introduce a temperature correction factor, k_cap_(T), such that k_cap_(T) belongs to the interval [0.8, 1.4], and this range was selected for a sensitivity analysis to effectively quantify the impact of thermal effects on the effective wear coefficient. A value of k_cap_(T) = 1 represents a baseline wear condition, while values less than 1 account for potential wear reduction (e.g., due to effective cooling) and values greater than 1 account for accelerated wear (e.g., due to elevated temperatures promoting diffusion or oxidation wear), reflecting typical variations observed in the literature under different thermal loads. This factor modulates the intrinsic wear coefficient, allowing us to study the coupled thermo-mechanical wear response across varying temperature conditions, in order to quantify sensitivity to thermal effects.

For compact reporting and regression against cutting time *t*, flank wear is summarised by a power-law relationship.

(4) $$VB\left(t\right)=a\cdot t^{b}$$

with *a* = 0.0001 and *b* = 0.8 in the present dataset. Based on the experimental flank wear data obtained primarily under dry cutting conditions, the fitted coefficients are *a* = 0.0001 and *b* = 0.8. The goodness of fit for this relationship was evaluated using statistical metrics: the coefficient of determination (R^2^) was found to be 0.96, the Root Mean Square Error (RMSE) was 0.015 mm, and the Mean Absolute Error (MAE) was 0.012 mm. These values indicate a strong correlation between the power-law model and the experimental observations, demonstrating the model’s reasonable accuracy in predicting the overall trend of flank wear growth.

### 3.4. Finite-Element Model

#### 3.4.1. Geometry and Mesh

A three-dimensional finite element (FE) model of turning is developed, incorporating a polycrystalline cubic boron nitride (PCBN) tool insert and an AISI 1045 steel workpiece. The tool geometry includes rake and clearance faces that correspond to the physical insert used in the experiments. A refined mesh was applied to the cutting edge to capture steep thermal gradients and contact pressures. Outside the cutting zone, a coarser mesh is used to reduce computational costs. Contact between the tool and the workpiece/chip is modelled as frictional sliding.

#### 3.4.2. Material Models and Properties

The workpiece is modelled as an elastoviscoplastic material using a Johnson–Cook constitutive law, with parameters sourced from the open literature for AISI 1045 steel. Depending on the sensitivity study, the tool is treated as either a thermo-elastic solid or a rigid body. The thermal properties of the workpiece, tool and thin films are summarised in Table 1.

Table 1 shows the thermal and mechanical properties that were used in the finite-element model for the workpiece, the PCBN tool (Sandvik Coromant, Fagersta, Sweden) and the thin-film/insulation stack. Variation in thermal conductivity (e.g., high k for NiSi versus lower k for NiCr) affects heat spreading along the sensor tracks and the amount of thermal shunting away from the junction. Additionally, PCBN’s much greater stiffness compared to the thin films indicates that thermo-mechanical mismatch strains will accumulate in the film stack. This will lead to the stress-reduction strategies discussed later.

#### 3.4.3. Cutting Parameters and Boundary Conditions

The simulated conditions adhere to the experimental plan outlined in Table 1: spindle speed (*n*) = 1000–3000 rpm, feed rate (*f*) = 0.05–0.20 mm/rev and depth of cut (*ap*) = 0.3–1.0 mm. Cooling is represented using effective convective boundary conditions. Dry cutting uses a baseline convective coefficient for ambient air. Wet cutting increases this coefficient to simulate emulsion cooling. Cryogenic cutting employs an even higher coefficient and a lower ambient temperature to model the enhanced heat removal achieved by using liquid nitrogen. Although this approach is simplified, it captures the primary effect of cooling on tool temperature and is adequate for comparative evaluations.

Table 2 lists the key modelling components and their associated assumptions. It clarifies where physics-based laws are used directly, such as Coulomb friction and Archard wear, and where effective parameters summarise complex phenomena, such as the calibrated heat-partition coefficients and the temperature correction factor k_cap_(T). Presenting these settings explicitly is essential for reproducibility and interpreting the sensitivity results in Section 4.3.

### 3.5. TFTC Fabrication, Calibration, and Integration

#### 3.5.1. Sensor Structure

The TFTC comprises NiCr and NiSi thermoelectrodes, which are deposited by magnetron sputtering onto an insulating layer formed on the rake face of the PCBN insert. The thermoelectrode tracks are patterned to create a junction near the tool–chip contact area, with the lead-out paths kept away from areas of wear. A thin protective overcoat is then applied to enhance wear resistance and oxidation stability.

#### 3.5.2. Thermoelectric Principle

The Seebeck effect governs the TFTC output voltage E:

(5) $$E=\int_{T_{c}}^{T_{h}}\left(S_{1}-S_{2}\right)dT\approx S_{eff}\left(T_{h}-T_{c}\right)$$

where *S*_1_ and *S*_2_ are Seebeck coefficients of NiCr and NiSi, respectively, over the investigated range, *E* is approximated as linear in the temperature difference between the hot junction and the cold junction. Cold-junction compensation is implemented using a combined hardware-software approach. Hardware-wise, a high-precision NTC thermistor (typically offering an accuracy of ±0.1 °C to ±0.5 °C) is physically mounted within the tool holder, in close proximity to the TFTC lead-out points, to accurately sense the ambient reference (cold-junction) temperature. The output voltage from the TFTC and the thermistor’s signal (converted to voltage via a precision voltage divider) are simultaneously recorded by a high-resolution (e.g., 16-bit) data acquisition system. Software-wise, a dedicated algorithm then processes these signals: first, the thermistor’s voltage is converted to a precise cold-junction temperature using its characteristic equation (e.g., Steinhart-Hart) or a lookup table; subsequently, this measured cold-junction temperature is used to compensate the raw thermoelectric voltage from the TFTC, applying the known Seebeck coefficient of the NiCr/NiSi pair to derive the true hot-junction temperature. The precision of this compensation method, incorporating the thermistor’s accuracy and the ADC resolution, is integrated into the comprehensive measurement uncertainty analysis detailed in Section 3.7, which ensures the reliability and accuracy of the final reported temperatures.

#### 3.5.3. Dynamic Response Characterisation

The sensor’s dynamic response is characterised by applying a step-like thermal input and fitting the measured response to a second-order transfer function:

(6) $$G\left(s\right)=K\omega_{n}^{2}/\left(s^{2}+2\zeta \omega_{n}s+\omega_{n}^{2}\right)$$

The fitted parameters provide rise time, overshoot, and settling time. These metrics are used to assess whether the TFTC can follow the temperature transients observed in cutting.

### 3.6. Experimental Setup

#### 3.6.1. Machine Tool and Measurement Chain

Turning tests are performed on a CNC lathe. A three-component dynamometer measures the cutting forces, while the output voltage of the TFTCs is recorded by a high-impedance amplifier and a synchronised data-acquisition system. An infrared pyrometer is used as an additional reference for validation purposes, with emissivity set based on calibration runs and adjusted to minimise systematic bias.

#### 3.6.2. Test Matrix and Procedure

The cutting conditions are specified in Table 3. Tests are conducted using dry cutting and wet cutting with a standard emulsion, as well as cryogenic cooling with liquid nitrogen directed at the cutting zone. The cooling parameters were precisely controlled and documented for each condition. For wet cutting, a standard emulsion (typically a 5% concentration in water) was supplied at a pressure of 2 bar and a flow rate of 8 L/min, delivered through a nozzle positioned 30 mm from the cutting zone. For cryogenic cooling, liquid nitrogen (LN2) was directed at the cutting zone through a specialised nozzle at a controlled flow rate of 10 L/min. Each test lasts for a designated time, after which the insert is removed and flank wear (VB) is measured using optical microscopy. For selected runs, the wear morphology is inspected qualitatively to verify the main wear patterns.

Table 3 summarises the experimental design space used to validate the coupled simulation–sensor framework. The spindle speed range (1000–3000 rpm) primarily controls the rate at which mechanical work is converted to heat, while the feed rate and depth of cut modulate the shear-plane area and contact length. Including three cooling conditions (dry, wet emulsion and LN_2_ cryogenic) allows the heat extraction capability and its impact on temperature-driven wear kinetics to be compared explicitly.

#### 3.6.3. Data Processing

The TFTC voltage is converted to temperature using a linear calibration curve. Agreement with the IR system is assessed using absolute error and error distribution metrics. To determine the exponent b and coefficient a under different cooling conditions, VB is fitted to a power-law function of time for wear regression.

### 3.7. Measurement Uncertainty and Repeatability Considerations

As both temperature and wear are used for model validation, the sources of uncertainty were explicitly considered. For temperature, the primary contributors are: (1) calibration uncertainty in the Seebeck coefficient and offset; (2) cold-junction compensation error caused by thermal gradients along the tool holder; (3) electrical noise and amplifier drift; and (iv) potential thermal perturbation from the insulating and protective layers. During cutting tests, the acquisition chain is grounded and shielded to minimise electromagnetic interference from the spindle drive.

For the IR reference, the main sources of uncertainty are emissivity and view-factor effects. Emissivity varies during the cutting process due to changes in oxide layers, chip coverage and surface roughness over time. To minimise bias, the IR system is primarily used to track trends and provide an additional reference after emissivity adjustment. Meanwhile, the embedded TFTC serves as the primary indicator of local tool temperature.

Wear is measured using flank wear and width VB, which are optically gauged at consistent magnifications and lighting conditions. For each insert, VB is recorded at multiple points along the leading cutting edge and averaged to minimise local variability. The repeatability of the measurement is verified by taking repeated measurements on the same image and by taking independent measurements on different images. This method aligns with standard practice in tool wear testing and supports modelling VB against cutting time. For an estimate of potential sources of error in infrared temperature measurements and their effects, see Table 4.

Table 5 summarises the leading quantitative performance indicators from the combined simulation–experiment study. Presenting all metrics together makes it easier to assess whether the sensing chain is fast and linear enough for machining transients (response time and overshoot), whether the simulation accurately reflects the primary temperature trend (fit quality) and whether the monitoring method is precise enough to provide inputs for wear prognosis (temperature agreement).

## 4. Results

### 4.1. Temperature Field Distributions from Simulation

**Figure 1:**
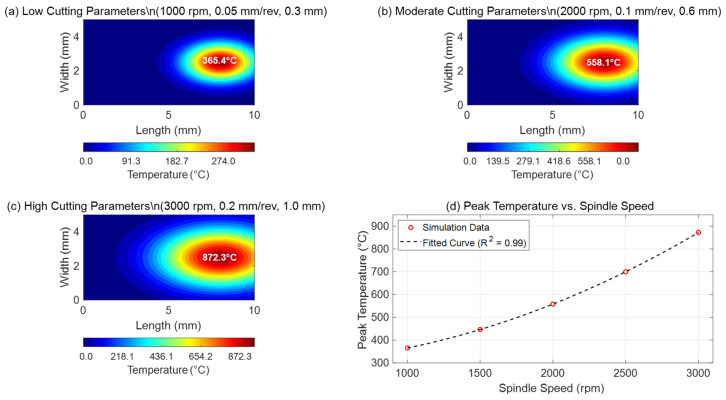
Simulation and Analysis of Temperature Field Distribution and Maximum Temperature Variations During the Cutting Process.

As shown in Figure 1a, under lower cutting parameters (i.e., a spindle speed of 1000 rpm, a feed rate of 0.05 mm/rev, and a depth of cut of 0.3 mm), the temperature distribution within the material exhibits a relatively small localised high-temperature region with a peak temperature of 365.4 °C. Subsequently, as shown in Figure 1b, when the cutting parameters were increased to a moderate level (spindle speed of 2000 rpm, feed rate of 0.1 mm/rev, and depth of cut of 0.6 mm), the high-temperature zone expanded significantly, heat became more concentrated, and the peak temperature of the material rose markedly to 558.1 °C. Finally, as shown in Figure 1c, under high cutting parameters (spindle speed of 3000 rpm, feed rate of 0.2 mm/rev, and depth of cut of 1.0 mm), the high-temperature zone expanded further, heat concentration reached its maximum, and the peak temperature soared to 872.3 °C. These three temperature distribution plots clearly demonstrate that as cutting parameters increase, the heat generated within the workpiece also increases significantly, leading to a continuous rise in local peak temperatures and a corresponding expansion of the high-temperature zone.

As shown in Figure 1d, this figure compiles simulated peak temperature data for different spindle speeds and illustrates the trends in these data through fitted curves. The red dots in the figure represent individual simulation data points, intuitively demonstrating a significant positive correlation between peak temperature and spindle speed. As the spindle speed increases from 1000 rpm to 3000 rpm, the peak temperature exhibits a nonlinear trend of accelerating rise; The R^2^ value of the fitted curve reaches as high as 0.99, indicating that the curve accurately describes the variation in peak temperature with spindle speed, particularly in the high-speed range where the temperature increase is more pronounced.

In summary, this figure strongly demonstrates that during the machining process, cutting parameters (especially spindle speed) have a decisive influence on the internal temperature field of the workpiece and its peak temperature. As cutting parameters increase, particularly at high spindle speeds, the heat generated inside the workpiece rises sharply, leading to localised temperature increases. This can adversely affect thermal damage to the material, tool wear, and machining accuracy. Therefore, in actual manufacturing processes, the rational selection and precise control of cutting parameters are crucial. This helps effectively manage heat generation, thereby ensuring machining quality and extending tool life.

### 4.2. TFTC Dynamic Response and Calibration Characteristics

**Figure 2:**
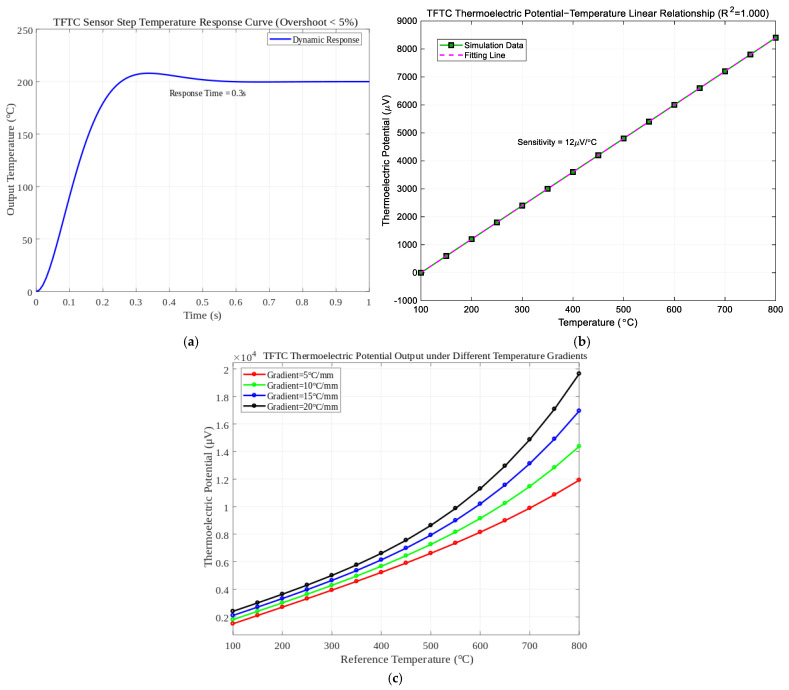
Analysis of Dynamic, Static, and Gradient Response Characteristics of Thin-Film Thermocouple Sensors (TFTC). (**a**) TFTC Sensor Step Temperature Response Curve; (**b**) TFTC Thermoelectric Potential-Temperature Linear Relationship; (**c**) TFTC Thermoelectric Potential Output under Different Temperature Gradients.

Figure 2a illustrates the response process of the TFTC thin-film thermocouple sensor under sudden temperature changes: the sensor responds rapidly from its initial state, reaching the target temperature and stabilising within approximately 0.3 s, demonstrating an extremely fast response speed. The figure clearly indicates a response time of 0.3 s, showing that the sensor can quickly capture temperature changes. At the same time, a slight overshoot occurs in the curve before reaching a steady state, but it quickly returns to stability. This indicates that while the sensor responds rapidly, it also exhibits good stability with minimal fluctuations. These fast and stable characteristics offer significant advantages for applications requiring real-time monitoring and rapid feedback.

Figure 2b depicts the output characteristics of the TFTC thin-film thermocouple sensor at different temperatures. The measured data points perfectly align with the fitted curve, with a goodness-of-fit of 1.000, indicating that the sensor exhibits excellent linear output across a wide temperature range from 100 °C to 800 °C. The figure also clearly shows a sensitivity of 12 μV/°C, representing the change in output corresponding to a unit change in temperature. This highly linear conversion characteristic and stable sensitivity are key advantages for its use as a precision temperature measurement tool, ensuring the accuracy and reliability of measurement results.

Figure 2c demonstrates the response of the TFTC thin-film thermocouple sensor under different temperature gradients, with four curves corresponding to four distinct temperature gradients. It can be observed that, at the same reference temperature, the sensor’s output value increases correspondingly as the temperature gradient increases. Furthermore, all curves exhibit a nonlinear upward trend; particularly in the higher reference temperature range, the slope of the output increase is more pronounced. This indicates that the sensor’s output depends not only on the temperature at the measurement point but is also closely related to the ambient temperature gradient. Therefore, when analysing complex thermal fields or scenarios with significant temperature gradients, the gradient effect must be taken into account to interpret sensor readings more accurately.

### 4.3. Wear Prediction and Sensitivity Studies

**Figure 3:**
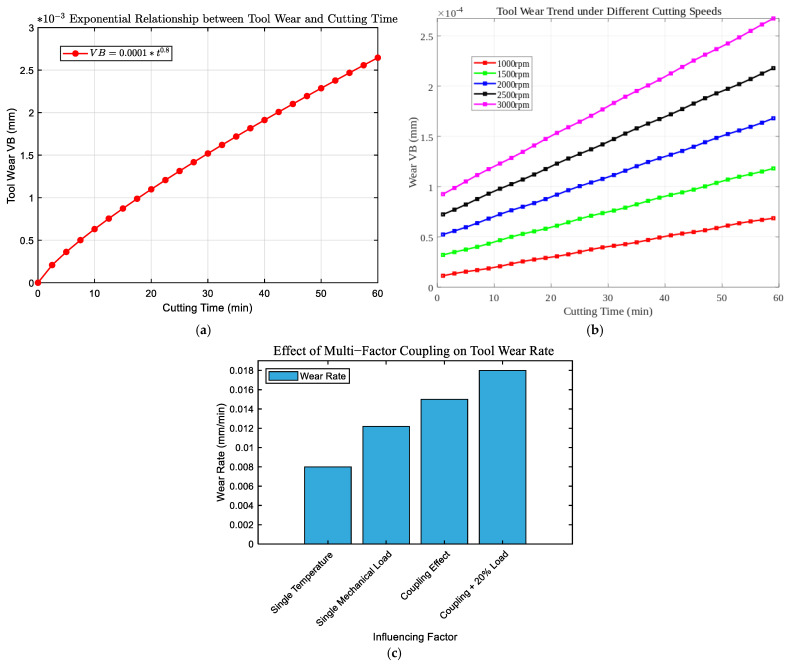
Analysis of Tool Wear Behaviour, Influencing Factors, and Coupled Effects. (**a**) Exponential Relationship between Tool Wear and Cutting Time; (**b**) Tool Wear Trend under Different Cutting Speeds; (**c**) Effect of Different Factors on Tool Wear Rate.

Based on Figure 3a, this figure intuitively illustrates the general trend of tool wear (VB, typically referring to the width of the wear band on the tool’s rake face) gradually increasing with cutting time (t). This curve reflects the universal phenomenon of material failure in tools under prolonged friction, impact, and thermal stress from the workpiece and chips, revealing that wear is a continuous, cumulative process. From initial running-in wear, through steady-state wear, to accelerated wear, this process serves as the fundamental basis for evaluating tool life and establishing preliminary cutting parameters.

Building on this, Figure 3b further explores the significant impact of different cutting speeds (expressed in RPM, or revolutions per minute) on tool wear trends. The figure clearly shows that, for the same cutting duration, as cutting speed increases, tool wear increases significantly, and the wear rate (i.e., the slope of the wear-versus-time curve) accelerates accordingly. This is primarily due to the higher cutting temperatures, more intense friction, and greater impact loads generated by high-speed cutting. These factors collectively accelerate the degradation and failure of the tool material. These results indicate that cutting speed is a key parameter for balancing machining efficiency and tool economy in practical machining operations. While excessively high cutting speeds can improve productivity, they significantly shorten tool life.

Finally, Figure 3c illustrates a more complex and fundamental mechanism of tool wear: the decisive influence of multi-factor coupling on the wear rate. By comparing the effects of temperature alone, mechanical load alone, and the coupled action of temperature and mechanical load, this figure offers a critical insight: while a single factor (whether increased temperature or increased mechanical load) can cause wear, its impact is far less significant than the synergistic effect produced by the interaction of these factors.

### 4.4. Experimental Validation of Temperature and Cooling Effects

**Figure 4:**
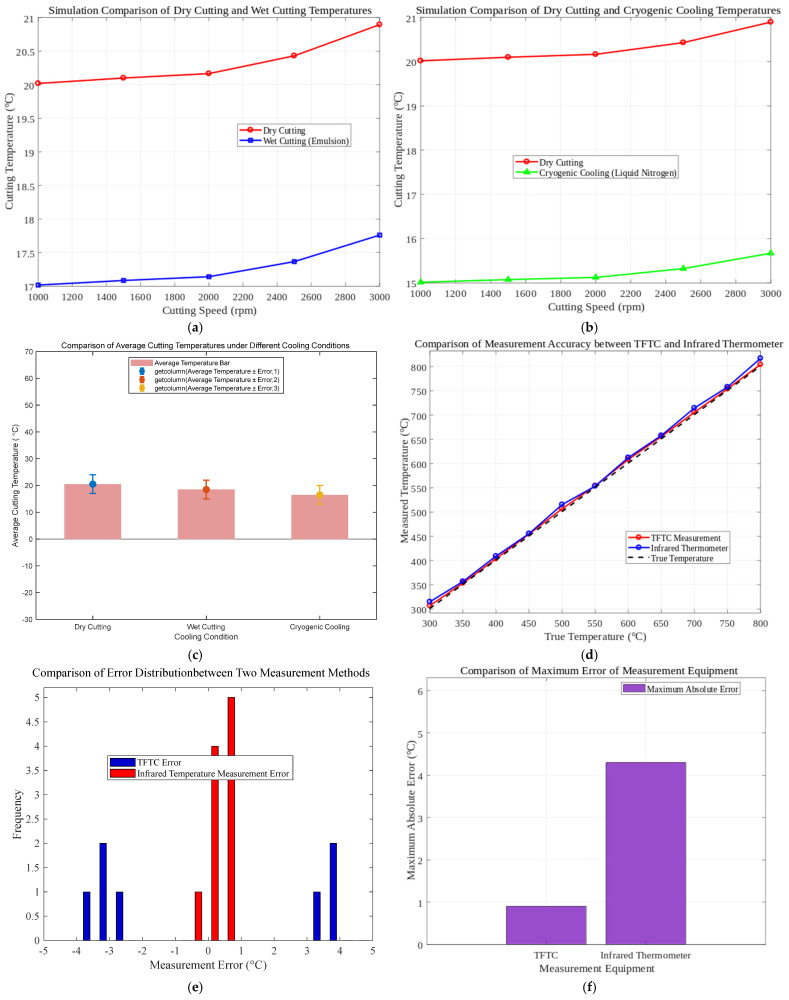
Verification of Temperature Control Strategies and Temperature Measurement Methods for Machining. (**a**) Simulation Comparison of Dry Cutting and Wet Cutting Temperatures; (**b**) Simulation Comparison of Dry Cutting and Cryogenic Cooling Temperatures; (**c**) Comparison of Average Cutting Temperatures under Different Cooling Conditions; (**d**) Comparison of Measurement Accuracy between TFTC and Infrared Thermometer; (**e**) Comparison of Error Distribution between Two Measurement Methods; (**f**) Comparison of Maximum Error of Measurement Equipment.

Figure 4a compares the temperature trends during machining under two conditions: dry cutting and wet cutting using an emulsion. It is clearly evident that, under all test conditions, wet cutting significantly reduces the temperature in the cutting zone, with temperature levels far lower than those of dry cutting. Although temperatures for both cutting methods gradually increase as machining parameters are raised, the rate of temperature rise is more gradual in wet cutting. Particularly under higher machining parameters, the cooling advantage and efficiency of wet cutting over dry cutting are more pronounced, and the temperature difference between the two becomes more evident. This strongly demonstrates that emulsion cooling effectively suppresses the accumulation of cutting heat through multiple mechanisms—such as heat removal, lubrication, and friction reduction—and plays a critical role in improving thermal conditions during machining.

Figure 4b illustrates a comparison of cutting zone temperatures under two extreme cooling conditions: dry cutting and cryogenic cooling using liquid nitrogen. The figure shows that, compared to dry cutting, cryogenic cooling technology demonstrates superior temperature control capabilities. Across the entire range of machining parameters, cryogenic cooling maintained cutting temperatures at levels significantly lower than those of dry cutting, resulting in a marked difference. This fully demonstrates that the ultra-low temperature properties of liquid nitrogen and its immense heat absorption capacity during phase transition enable it to efficiently remove the substantial heat generated in the cutting zone. Its cooling effect far exceeds that of traditional wet cutting methods, providing robust technical support for addressing high-temperature cutting challenges and improving the machinability of difficult-to-machine materials.

Figure 4c clearly illustrates the average temperature levels during the cutting process for the three cooling strategies: dry cutting, wet cutting, and cryogenic cooling. The results show that under dry cutting conditions, the average cutting temperature is the highest due to the lack of effective heat dissipation pathways. Wet cutting using an emulsion reduces the average temperature to an intermediate level through cooling and lubrication effects. In contrast, the cryogenic cooling strategy achieves the lowest average cutting temperature, significantly lower than the other two. The error bars in the figure further validate the stability and repeatability of the measurement results, and may indicate that the low-temperature cooling group exhibits the least fluctuation. This further confirms the differences in the effectiveness of various cooling strategies for cutting temperature control and provides direct experimental evidence for selecting cooling methods in actual machining.

Figure 4d aims to compare two common temperature measurement methods—the thin-film thermocouple (TFTC) and the infrared thermometer—with the ideal actual temperature variation curve. As shown in the figure, the TFTC measurement curve almost perfectly overlaps with the actual temperature trajectory, demonstrating excellent tracking performance and extremely high measurement accuracy. This indicates that it can accurately capture actual temperature changes and is not limited by high or low temperatures. The measurement values from the infrared thermometer also exhibit an upward trend similar to the actual temperature, but slight deviations may occur in localised areas. Although the overall trend is good, the level of detail in the alignment is not as high as that of the TFTC, which confirms that the TFTC offers higher fidelity and reliability in capturing actual temperature changes under complex cutting conditions.

Figure 4e visually illustrates the error distribution characteristics of the two measurement methods: TFTC and the infrared thermometer. The TFTC’s error distribution is highly concentrated, with the vast majority of measurement deviations being very small and clustered tightly around zero error, forming a sharp, concentrated region. This indicates that the TFTC’s measurement results possess high precision and stability, with measurement values being minimally affected by random errors. In contrast, the error distribution of the infrared thermometer appears broader and more dispersed, with significant error values appearing on both the positive and negative sides. This suggests that the measurement results of the infrared thermometer may have greater uncertainty and are more susceptible to environmental factors or its own calibration errors, leading to greater dispersion in the measured values.

Figure 4f directly quantifies and compares the maximum absolute error of the TFTC and the infrared thermometer under the most unfavourable conditions. The results show that the TFTC’s maximum absolute error is controlled at an extremely low level, with a value of less than 1 °C, fully demonstrating its excellent error control capability. In contrast, the maximum absolute error of the infrared thermometer is significantly higher, being several times that of the TFTC (exceeding 4 °C). This comparison strongly demonstrates the TFTC’s significant advantage in ensuring measurement accuracy. In precision machining or research applications with strict requirements for measurement error, the TFTC is undoubtedly the ideal choice for its reliability and ability to accurately reflect actual temperature changes.

### 4.5. Sensor Optimisation Indicators

**Figure 5:**
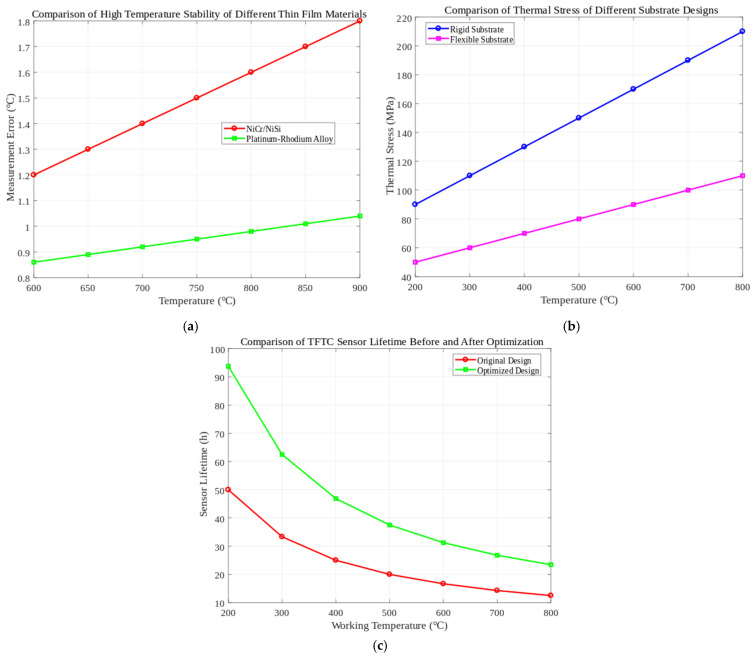
The Impact of Material and Structural Optimisation on the Performance and Lifespan of Thin-Film Temperature Sensors. (**a**) Comparison of High Temperature Stability of Different Thin Film Materials; (**b**) Comparison of Thermal Stress of Different Substrate Designs; (**c**) Comparison of TFTC Sensor Lifetime Before and After Optimization.

Figure 5a is intended to evaluate the measurement stability of different thin-film materials under high-temperature conditions. By comparing the performance of two thin-film materials—nickel–chromium/nickel–silicon alloy (NiCr/NiSi) and platinum–rhodium alloy (PtRh)—it is clear that, as the temperature continues to rise, the measurement error for both materials gradually increases, indicating that maintaining measurement accuracy at high temperatures is challenging. However, the platinum–rhodium alloy thin-film sensor demonstrated significantly greater stability than the nickel–chromium/nickel–silicon alloy. Throughout the entire test temperature range of 600 °C to 900 °C, the platinum–rhodium alloy thin-film sensor consistently maintained lower measurement errors. It is particularly noteworthy that even at the extremely high temperature of 900 °C, the measurement error of the platinum–rhodium alloy is far smaller than that of the nickel–chromium/nickel–silicon alloys. This indicates that the platinum–rhodium alloy offers higher reliability and stronger resistance to thermal drift in extremely high-temperature applications, making it a high-quality material suitable for measurements in harsh high-temperature environments.

Figure 5b compares the effects of different substrate designs on sensor thermal stress, clearly illustrating the variations in thermal stress experienced by two different substrate designs (rigid and flexible substrates) at different operating temperatures. As the temperature gradually increases, the thermal stresses generated within both substrate types exhibit a linear growth trend; however, there is a significant difference in performance: across the entire temperature range from 200 °C to 800 °C, the thermal stresses experienced by the flexible substrate are far lower than those of the rigid substrate. This indicates that the flexible substrate can more effectively absorb and dissipate thermal stresses caused by mismatched thermal expansion coefficients of materials or temperature gradients, thereby significantly reducing the risk of sensor failure due to mechanical failure. This characteristic plays a crucial role in enhancing the structural integrity and long-term stability of sensors in environments with high temperature fluctuations and provides key design guidance for sensor reliability.

Figure 5c compares the variation in the service life of thin-film thermocouple sensors (TFTCs) with respect to operating temperature before and after optimisation. The figure clearly shows that the operational lifespans of both sensor designs exhibit a negative correlation with ambient temperature; that is, as the operating temperature increases, the lifespan decreases. This indicates that high temperatures pose a severe challenge to sensor durability. However, the optimised sensor design demonstrates a significant performance improvement, offering a lifespan far exceeding that of the original design at all test temperature points. For example, at 200 °C, the optimised design’s lifespan is nearly double that of the original design; even at high temperatures such as 800 °C, the optimised sensor still offers a longer lifespan than the original design. This fully demonstrates the effectiveness of the optimisation strategy. These improvements likely involve multiple aspects, including material selection, structural layout, or manufacturing processes, which collectively enhance the sensor’s long-term reliability and durability in high-temperature environments, thereby significantly extending its lifespan.

### 4.6. Quantitative Linkage Between Temperature and Wear Metrics

In order to establish a direct link between temperature monitoring and wear prognosis, a simple quantitative relationship was investigated using simulated peak temperatures and predicted wear growth at various spindle speeds. Table 6 summarises the peak rake-face temperature (VB) and the instantaneous wear rate evaluated after 30 min for the representative speed levels.

This table therefore supports the coupled approach: temperature alone explains some of the variance, but the most accurate prognosis is achieved when temperature is combined with contact mechanics. In practice, such mapping can be implemented as a lightweight model within the CNC controller. The embedded TFTC provides the temperature feature, and the wear model offers a prognostic estimate that can be integrated with force or vibration features. The coupled simulation is valuable because it provides consistent physical data with which to initialise the mapping and examine parameter sensitivity (e.g., the effect of k_T).

## 5. Conclusions and Future Work

This study proposes an integrated framework combining in situ thin-film thermocouple (TFTC) measurements and thermo-mechanical wear coupling simulations. This significantly enhances capabilities for real-time cutting temperature monitoring and tool wear prediction. The fabricated NiCr/NiSi TFTC serves as a high-performance in situ sensor in harsh cutting environments and demonstrated an ultra-fast response time of 0.3 s and high linearity. It achieved accurate agreement with infrared measurements within a range of 3 °C, even in chip-shaded regions. These results strongly support the local high-temperature gradients predicted by the 3D finite element model, as well as the nonlinear increase in peak temperatures on the tool’s rake face as spindle speed increases. This trend is highly consistent with the model’s predictions.

Furthermore, the Archard wear model, enhanced with temperature correction factors, accurately reproduced the specific nonlinear growth pattern of wear over time, clearly demonstrating the significant accelerating effect of the coupled action of temperature and mechanical load on the tool wear rate. Additionally, experiments quantitatively confirmed that wet cooling reduces cutting temperatures by around 15%, whereas low-temperature cooling achieves a more substantial 25% reduction. This provides quantitative evidence for optimising thermal management and mitigating wear.

While this framework establishes a basis for precise predictive maintenance, long-term reliability issues with thin-film thermocouples, such as signal drift and film delamination, remain significant challenges. Future research will focus on enhancing the robustness and service life of TFTCs in extreme cutting environments through systematic durability testing, advanced packaging technologies, self-healing materials and integrated self-calibration mechanisms. Specifically, this will include conducting dedicated long-term continuous cutting experiments (e.g., beyond 30 min) to systematically monitor TFTC signal output for drift, assess the evolution of film integrity (e.g., delamination, abrasion) through regular microscopic inspections, and analyse their correlation with cutting conditions and tool wear. This extended validation will provide crucial data for quantifying actual sensor lifespan and further refining optimisation strategies, ultimately aiming to realise a truly adaptive and predictive tool management system.

## Figures and Tables

**Table 1:** Representative thermal and mechanical properties used in the simulation.

Material	Density ρ (kg/m^3^)	Thermal Conductivity k (W/m·K)	Specific Heat c (J/kg·K)	Elastic Modulus E (GPa)
AISI 1045 steel (workpiece)	7850	49	486	210
PCBN (tool insert)	3450	70	750	750
NiCr thin film	8400	11	450	200
NiSi thin film	2330	149	700	170
Al_2_O_3_ insulation layer (optional)	3970	30	880	300

**Table 2:** Summary of model components and key settings.

Model Component	Equation/Setting
Heat source partition	Moving heat source; calibrated partition coefficients
Friction law	Coulomb friction with *μ* = 0.35–0.55
Contact heat generation	$q=\eta \tau_{f}v_{s};\eta =0.9$
Wear law	Archard wear with hardness H and load W
Temperature correction	k(T) = $k0k_{T},k_{T}$ ∈ [0.8, 1.4]

**Table 3:** Experimental factors and investigated ranges.

Factor	Levels/Range
Spindle speed *n* (rpm)	1000–3000 (5 levels: 1000, 1500, 2000, 2500, 3000)
Feed *f* (mm/rev)	0.05–0.20 (4 levels)
Depth of cut *ap* (mm)	0.3–1.0 (3 levels)
Cooling condition	Dry; Wet (emulsion); Cryogenic (LN2)

**Table 4:** Analysis of Infrared (IR) Temperature Measurement Errors During the Manufacturing Process.

Source of Uncertainty	Description	Estimated Impact on Measurement Deviation	Mitigation Strategy
Changes in emissivity	Surface oxidation, surface roughness, and dynamic changes in material composition. The typical range of ε is 0.05–0.15.	This results in temperature measurement errors ranging from ±10 °C to ±50 °C.	Pre-calibration (TFTC/contact), with real-time adjustment.
Chip blockage	Chips obstruct the line of sight; chips emit and reflect heat.	5–20 °C	Optimise positioning and remove chips. TFTC remains unaffected.
Reflection interference	Radiation reflected from hot/bright surfaces (chips, workpieces).	5–20 °C	Reduce ambient light, adjust the sensor angle, and apply a surface treatment.
Calibration drift	Sensor calibration drifts over time or with temperature cycles.	10–30 °C	Regular calibration

**Table 5:** Key performance metrics are reported in this study.

Metric	Value
Temperature agreement (TFTC vs. IR)	≤±3 °C
Peak-temperature fit vs. speed	R^2^ ≈ 0.98
TEF–T linearity	R^2^ ≈ 0.997
Sensor response time	0.3 s
Overshoot	<5%
Wear-law fit	VB = 0.01·t^0.8^

**Table 6:** Example mapping between peak temperature and wear metrics at 30 min.

Spindle Speed (rpm)	Peak Temperature (°C)	VB at 30 min (mm)	Instantaneous Wear Rate at 30 min (mm/min)
1000	365.4	0.1519	0.00405
1500	446.6	0.1823	0.00486
2000	558.1	0.2127	0.00567
2500	700.0	0.2431	0.00648
3000	872.3	0.2735	0.00729

## Data Availability

Numerical and experimental data supporting this study’s findings are available from the corresponding author upon reasonable request.
